# Molecular mechanisms of azole resistance in *Candida* bloodstream isolates

**DOI:** 10.1186/s12879-019-3672-5

**Published:** 2019-01-17

**Authors:** Jocelyn Qi-Min Teo, Shannon Jing-Yi Lee, Ai-Ling Tan, Robyn Su-May Lim, Yiying Cai, Tze-Peng Lim, Andrea Lay-Hoon Kwa

**Affiliations:** 1 Department of Pharmacy, Singapore General Hospital, Blk 8 Level 2, Outram Road, Singapore, 169608 Singapore; 2 Saw Swee Hock School of Public Health, National University of Singapore, 12 Science Drive 2, #10-01, Singapore, 117549 Singapore; 3 Department of Microbiology, Singapore General Hospital, Outram Road, Singapore, 169608 Singapore; 4 Department of Pharmacy, National University of Singapore, 18 Science Drive 4, Singapore, 117543 Singapore; 5 Singhealth Duke-NUS Pathology Academic Clinical Programme, 8 College Road, Level 4, Singapore, 169857 Singapore; 6 Singhealth Duke-NUS Medicine Academic Clinical Programme, 8 College Road, Level 4, Singapore, 169857 Singapore; 7 Emerging Infectious Diseases, Duke-National University of Singapore Medical School, 8 College Rd, Singapore, 169857 Singapore; 8 Present address: Program in Health Services and Systems Research, Duke-National University of Singapore Medical School, 8 College Rd, Singapore, 169857 Singapore

**Keywords:** Candida, Antifungal resistance, Genomics

## Abstract

**Background:**

Antifungal resistance rates are increasing. We investigated the mechanisms of azole resistance of *Candida* spp. bloodstream isolates obtained from a surveillance study conducted between 2012 and 2015.

**Methods:**

Twenty-six azole non-susceptible *Candida* spp. clinical isolates were investigated. Antifungal susceptibilities were determined using the Sensititre YeastOne® YO10 panel. The *ERG11* gene was amplified and sequenced to identify amino acid polymorphisms, while real-time PCR was utilised to investigate the expression levels of *ERG11*, *CDR1*, *CDR2* and *MDR1*.

**Results:**

Azole cross-resistance was detected in all except two isolates. Amino acid substitutions (A114S, Y257H, E266D, and V488I) were observed in all four *C. albicans* tested. Of the 17 *C. tropicalis* isolates, eight (47%) had *ERG11* substitutions, of which concurrent observation of Y132F and S154F was the most common. A novel substitution (I166S) was detected in two of the five *C. glabrata* isolates. Expression levels of the various genes differed between the species but *CDR1* and *CDR2* overexpression appeared to be more prominent in *C. glabrata*.

**Conclusions:**

There was interplay of various different mechanisms, including mechanisms which were not studied here, responsible for azole resistance in *Candida* spp in our study.

## Background

*Candida* bloodstream infections are an important healthcare issue known to be associated with high morbidity and mortality. There have been increasing reports of antifungal resistance. We have previously reported decreasing azole susceptibilities in our hospital, particular in *Candida tropicalis*. More than 20% of *C. tropicalis* were non-susceptible to fluconazole [1]. There are various mechanisms mediating azole resistance. It has been suggested that molecular mechanisms such as presence of mutations may be a predictive marker of clinical failure in *Candida* infections [2]. Whilst this has been established for echinocandin resistance, azole resistance mechanisms are not as well studied, particularly for non-albicans species. Elucidation of these mechanisms is crucial to make progress in understanding and treating invasive *Candida* infections.

## Methods

In this study, we characterised the molecular mechanisms of azole resistance in 26 fluconazole non-susceptible *Candida* bloodstream isolates. These isolates were identified from a retrospective surveillance study conducted at a major regional tertiary referral hospital between 2012 and 2015 [1]. In brief, non-duplicate *Candida* bloodstream isolates from all adult inpatients (at least 21 years old) with temporally-related clinical signs and symptoms of infection admitted to the hospital during the study period were included. Antifungal susceptibility testing was performed using Sensititre YeastOne® YO10 panel (Trek Diagnostics System, West Sussex, England) according to manufacturer’s recommendations. Minimum inhibitory concentrations were interpreted according to the current species-specific clinical breakpoints provided by the Clinical and Laboratory Standards Institute (CLSI) M27-S4 document or epidemiological cut-off values (ECV), where CLSI breakpoints were unavailable [3, 4]. For *Candida albicans* and *C. tropicalis*, isolates meeting the susceptible-dose-dependent (SDD) and resistant criteria were included, whereas only resistant *Candida glabrata* were included in this study. A total of 26 fluconazole non-susceptible isolates [*C. albicans -* 4/62 (6%); *C. glabrata -* 5/82 (6%); *C. tropicalis -* 17/78 (22%); *C. parapsilosis* - 0/35 (0%)] were identified from 257 *Candida* spp. isolates included in the surveillance study.

*ERG11*, *CDR1, CDR2* and *MDR1* gene expression were quantified in triplicates using real-time reverse transcription-PCR (RTPCR) with total RNA extracted from exponential-phase yeast peptone dextrose broth cultures on a CFX96 Real-Time PCR Detection System (Bio-Rad, USA). The primers used were adopted from previous publications [5–11], except for *C. glabrata CDR1* gene [F – TGGTGTTGCTAATGTCGCCA, R – GTCCCAAGTACTCGCCACAA] and *C. glabrata ERG11* gene [F – CCACCCATTGCACTCTTTGT, R – AGAACGTGGTAGTCCCTTGG]. Quantification of target genes was normalised to the level of *ACT1*, an endogenous reference gene. Relative gene expression was calculated as the fold change in expression of the isolates compared to the respective ATCC reference strains (*C. albicans* ATCC 90028, *C. glabrata* ATCC 2950, *C. tropicalis* ATCC 750). A fold increase of 3 times was considered to be an overexpression of the target gene. The *ERG11* gene was amplified and sequenced to identify amino acid mutations by comparing with reference wild-type GenBank sequences (*C. albicans* – X13296; *C. tropicalis* – M23673; *C. glabrata* – L40389).

## Results

The susceptibility profiles of the isolates are displayed in Table 1. Cross-resistance to all azoles was observed in all isolates except for one *C. albicans* (CW138) and two *C. glabrata* (CW262 and CW378) isolates. All isolates retained susceptibility to other anti-fungals including anidulafungin, caspofungin, micafungin and amphotericin B (data not shown). In *C. albicans*, all isolates showed non-synonymous homozygous *ERG11* substitutions which included three distinct substitutions (A114S, Y257H and E266D). I166S substitutions were detected in two of the six *C. glabrata* isolates. Of the 17 *C. tropicalis* isolates, eight (47%) had *ERG11* substitutions. The most common substitutions were the concurrent observation of Y132F and S154F, which occurred primarily in resistant isolates with fluconazole MICs ≥8 μg/mL. Only two of the eight *ERG11* substitutions were homozygous, and there does not appear to be any correlation of the type of substitutions with MICs. The *ERG11* substitutions observed in all of the *Candida* spp. have been previously reported in literature except for I166S.

**Table 1:** Molecular characteristics of clinical fluconazole non-susceptible *Candida* spp. blood isolates

Isolate reference		MIC, ug/mL			Gene expression (fold increase)				Erg11p amino acid substitution(s)
		FLC	VRC	POS	*ERG11*	*CDR1*	*CDR2*	*MDR1*	
*C. albicans*	CW138	4 (SDD)	0.12 (S)	0.25 (NWT)	0.41	1.60	**9.01**	0.18	A114S, Y257H
	CW357	4 (SDD)	0.25 (SDD)	0.25 (NWT)	0.18	1.08	**5.79**	0.15	A114S, Y257H
	CW241	128 (R)	4 (R)	1 (NWT)	0.58	**4.49**	**122.50**	**141.28**	A114S, Y257H
	CW216	128 (R)	≥8 (R)	≥8 (NWT)	0.15	0.79	**3.32**	**4.86**	E266D, V488I
*C. glabrata*	CW193	64 (R)	4 (NWT)	≥8 (NWT)	0.46	**23.12**	**23.24**	N.A.	None
	CW262	64 (R)	0.25 (WT)	0.5 (WT)	1.08	**22.45**	**7.13**	N.A.	I166S
	CW378	64 (R)	4 (NWT)	2 (WT)	0.56	**4.02**	**8.08**	N.A.	None
	CW088	≥256 (R)	4 (NWT)	≥8 (NWT)	1.10	**19.78**	**14.55**	N.A.	None
	CW404	≥256 (R)	≥8 (NWT)	≥8 (NWT)	0.29	**18.70**	**6.92**	N.A.	I166S
*C. tropicalis*	CW190	4 (SDD)	0.25 (SDD)	0.5 (NWT)	0.39	**6.11**	N.A.	0.47	None
	CW219	4 (SDD)	0.5 (R)	0.5 (NWT)	0.95	1.56	N.A.	0.77	None
	CW361	4 (SDD)	0.5 (R)	0.25 (NWT)	2.38	0.27	N.A.	0.01	None
	CW395	4 (SDD)	0.5 (R)	0.5 (NWT)	0.45	0.63	N.A.	**3.27**	None
	CW071	8 (R)	0.25 (SDD)	0.5 (NWT)	0.11	1.28	N.A.	**30.53**	None
	CW018	16 (R)	0.25 (SDD)	0.25 (NWT)	0.61	2.75	N.A.	**23.42**	Y132F, S154F
	CW107	16 (R)	1 (R)	0.5 (NWT)	0.36	0.09	N.A.	**7.12**	Y132F, S154F
	CW178	16 (R)	0.5 (R)	1 (NWT)	2.83	**3.81**	N.A.	0.64	None
	CW385	16 (R)	0.5 (R)	1 (NWT)	0.12	**4.43**	N.A.	2.93	None
	CW263	64 (R)	4 (R)	0.12 (NWT)	**4.45**	1.95	N.A.	1.44	Y132F, S154F
	CW386	128 (R)	2 (R)	4 (NWT)	1.36	0.68	N.A.	1.33	None
	CW065	≥256 (R)	4 (R)	0.5 (NWT)	1.04	1.30	N.A.	**23.07**	Y132F, S154F
	CW067	≥256 (R)	≥8 (R)	1 (NWT)	0.11	0.93	N.A.	**7.04**	Y132F, S154F, F145 L
	CW192	≥256 (R)	≥8 (R)	1 (NWT)	1.36	0.76	N.A.	0.52	Y132F, S154F
	CW242	≥256 (R)	≥8 (R)	1 (NWT)	0.50	**6.10**	N.A.	1.21	Y132F, S154F
	CW266	≥256 (R)	4 (R)	0.25 (NWT)	0.27	**10.08**	N.A.	1.59	None
	CW271	≥256 (R)	≥8 (R)	1 (NWT)	**7.47**	1.30	N.A.	0.31	Y132F, S154F

*FLC* Fluconazole, *VRC* Voriconazole, *POS* Posaconazole, *S* Susceptible, *SDD* Susceptible dose-dependent, *R* Resistant, *WT* Wild-type, *NWT* Non wild-type, values in bold represent gene overexpression; underlined values represent heterozygous substitutions

Among the different gene targets, it appeared that *ERG11* expression levels were mostly similar compared to the respective wild-type reference strains. *CDR2* expression was consistently elevated in fluconazole non-susceptible *C. albicans.* In the two resistant isolates with MIC 128 μg/mL, *MDR1* was also up-regulated. *CDR1* and *CDR2* co-expression was observed in all *C. glabrata* isolates. Gene overexpression was not consistent among *C. tropicalis* isolates – there were five isolates with *CDR1* overexpression and six isolates with *MDR1* overexpression. All *C. tropicalis* isolates only had overexpression of a single gene target. Interestingly, there were three *C. tropicalis* isolates with no *ERG11* mutations or any gene up-regulation.

## Discussion

In this study, we evaluated the molecular mechanisms associated with azole resistance in various *Candida* species in our institution. Identification of antifungal susceptibilities through phenotypic methods such as MIC testing is often limited by the length of time required. Furthermore, current fungal MIC breakpoint interpretations are not supported by robust clinical data and are not predictive of clinical success/failure. Therefore, there is interest in identifying genotypic markers which could be rapidly identified for use in clinical prediction. Various previous studies have investigated different mechanisms of azole resistance in *Candida* species [5, 12–14]. Some of these studies have identified key *ERG11* substitutions which are associated with azole resistance e.g. Y132F, S154F [8, 15] and suggested that these mutations could be potential predictive markers of azole resistance.

In our context, it appeared that there was an interplay of various different mechanisms, including mechanisms which were not studied here, responsible for azole resistance in *Candida* spp*. ERG11* mutations were commonly detected in *C. albicans*, whereas the role of overexpression of azoles efflux pumps appeared to be more prominent in *C. albicans* (*CDR1*) and *C. glabrata* (*CDR1*, *CDR2*). In *C. tropicalis*, presence of Y132F and S154F substitutions was unable to explain the mechanisms of majority of our isolates. Less than half of the azole-resistant *C. tropicalis* harboured these amino acid substitutions. This was in contrast to the high frequency identified in another local study where > 90% of the isolates had Y132F and S154F substitutions [15]. Likewise, in another study, these mutations accounted for 95% of the fluconazole-resistant *C. tropicalis* [16].

Our study was limited by the small sample size although we had included all azole-resistant bloodstream isolates between 2012 and 2015. In addition, we did not perform further functional verification of the *ERG11* mutations and homology modelling experiments, therefore the clinical significance of I166S amino acid substitution in *C. glabrata* remains to be validated.

## Conclusions

In conclusion, our results indicated that the mechanisms mediating azole resistance in our isolates are heterogeneous. There were isolates with unidentified resistance mechanisms warranting further exploration. Moving ahead, the use of more advanced molecular technologies such as next-generation sequencing might be considered for an in-depth molecular characterisation of azole-resistant *Candida* spp to aid the identification of potential resistance markers.

## Abbreviations

ATCC: American Type Culture Collection
CLSI: Clinical and Laboratory Standards Institute
ECV: Epidemiological cut-off values
PCR: Polymerase chain reaction
RTPCR: Real-time reverse-transcription polymerase chain reaction

## References

[1] Teo JQ, Candra SR, Lee SJ, Chia SY, Leck H, Tan AL, Neo HP, Leow KW, Cai Y, Ee RP (2017). Candidemia in a major regional tertiary referral hospital - epidemiology, practice patterns and outcomes. Antimicrob Resist Infect Control.

[2] Shields RK, Nguyen MH, Press EG, Kwa AL, Cheng S, Du C, Clancy CJ (2012). The presence of an FKS mutation rather than MIC is an independent risk factor for failure of echinocandin therapy among patients with invasive candidiasis due to Candida glabrata. Antimicrob Agents Chemother.

[3] CLSI (2012). Reference Method for Broth Dilution Antifungal Susceptibility Testing of Yeasts; Fourth Informational Supplement. CLSI document M27-S4.

[4] Canton E, Peman J, Iniguez C, Hervas D, Lopez-Hontangas JL, Pina-Vaz C, Camarena JJ, Campos-Herrero I, Garcia-Garcia I, Garcia-Tapia AM (2013). Epidemiological cutoff values for fluconazole, itraconazole, posaconazole, and voriconazole for six Candida species as determined by the colorimetric Sensititre YeastOne method. J Clin Microbiol.

[5] Vandeputte P, Larcher G, Berges T, Renier G, Chabasse D, Bouchara JP (2005). Mechanisms of azole resistance in a clinical isolate of Candida tropicalis. Antimicrob Agents Chemother.

[6] Chau AS, Mendrick CA, Sabatelli FJ, Loebenberg D, McNicholas PM (2004). Application of real-time quantitative PCR to molecular analysis of Candida albicans strains exhibiting reduced susceptibility to azoles. Antimicrob Agents Chemother.

[7] Mane A, Vidhate P, Kusro C, Waman V, Saxena V, Kulkarni-Kale U, Risbud A (2016). Molecular mechanisms associated with fluconazole resistance in clinical Candida albicans isolates from India. Mycoses.

[8] Jiang C, Dong D, Yu B, Cai G, Wang X, Ji Y, Peng Y (2013). Mechanisms of azole resistance in 52 clinical isolates of Candida tropicalis in China. J Antimicrob Chemother.

[9] Caudle KE, Barker KS, Wiederhold NP, Xu L, Homayouni R, Rogers PD (2011). Genomewide expression profile analysis of the Candida glabrata Pdr1 regulon. Eukaryot Cell.

[10] Li QQ, Skinner J, Bennett JE (2012). Evaluation of reference genes for real-time quantitative PCR studies in Candida glabrata following azole treatment. BMC Mol Biol.

[11] Chen LM, Xu YH, Zhou CL, Zhao J, Li CY, Wang R (2010). Overexpression of CDR1 and CDR2 genes plays an important role in fluconazole resistance in Candida albicans with G487T and T916C mutations. J Int Med Res.

[12] Henry KW, Nickels JT, Edlind TD (2000). Upregulation of ERG genes in Candida species by azoles and other sterol biosynthesis inhibitors. Antimicrob Agents Chemother.

[13] Sanguinetti M, Posteraro B, Fiori B, Ranno S, Torelli R, Fadda G (2005). Mechanisms of azole resistance in clinical isolates of Candida glabrata collected during a hospital survey of antifungal resistance. Antimicrob Agents Chemother.

[14] Eddouzi J, Parker JE, Vale-Silva LA, Coste A, Ischer F, Kelly S, Manai M, Sanglard D (2013). Molecular mechanisms of drug resistance in clinical Candida species isolated from Tunisian hospitals. Antimicrob Agents Chemother.

[15] Chew KL, Cheng JWS, Jureen R, Lin RTP, Teo JWP (2017). ERG11 mutations are associated with high-level azole resistance in clinical Candida tropicalis isolates, a Singapore study. Mycoscience.

[16] Fan X, Xiao M, Zhang D, Huang JJ, Wang H, Hou X, Zhang L, Kong F, Chen SC, Tong ZH, et al. Molecular mechanisms of azole resistance in Candida tropicalis isolates causing invasive candidiasis in China. Clin Microbiol Infect. 2018. 10.1016/j.cmi.2018.11.007. Epub ahead of print. 10.1016/j.cmi.2018.11.007 30472420

